# Differential regulation of ATP hydrolysis of RIG-I-like receptors by transactivation response RNA-binding protein

**DOI:** 10.1042/BSR20222152

**Published:** 2023-05-05

**Authors:** Benyapa Chunhaphinyokul, Emi Hosokai, Masahiko Miyamoto, Akihiko Komuro

**Affiliations:** Department of Biochemistry, Faculty of Pharmaceutical Sciences, Niigata University of Pharmacy and Applied Life Sciences, Niigata 956-8603, Japan

**Keywords:** ATPase, host-pathogen interactions, innate immunity, interferons, RNA-binding proteins

## Abstract

Retinoic acid inducible gene (RIG)-I-like receptors (RLRs), including RIG-I, melanoma differentiation associated-5 (MDA5), and laboratory of genetics and physiology 2 (LGP2), play pivotal roles in viral RNA sensing to initiate antiviral interferon (IFN) responses.

We previously reported that an RNA-silencing regulator, transactivation response RNA-binding protein (TRBP), up-regulates MDA5/LGP2-mediated IFN responses through interaction with LGP2. Here, we aimed to investigate the mechanism underlying the TRBP-mediated up-regulation of IFN response.

Data indicated that phosphomimetic TRBP showed a modest effect, whereas the nonphosphorylated form exhibited hyperactivity in enhancing *Cardiovirus*-triggered IFN responses. These results suggest that encephalomyocarditis virus (EMCV) attenuates the TRBP-mediated IFN response via TRBP phosphorylation, since EMCV infection activates the kinase responsible for TRBP phosphorylation for virus replication.

Furthermore, we found that TRBP-mediated up-regulation of IFN response required the ATP hydrolysis and RNA binding of LGP2. TRBP enhanced RNA-dependent ATP hydrolysis by LGP2 but not that by RIG-I or MDA5. Nonphosphorylated TRBP exhibited higher levels of activity than phosphomimetic TRBP did, suggesting its possible involvement in the mechanism underlying the up-regulation of IFN response. TRBP activated the ATP hydrolysis of LGP2 and RIG-I, but not that of MDA5, in the absence of RNA. Collectively, we showed that TRBP differentially regulated RLR-mediated ATP hydrolysis. Further elucidation of the mechanism underlying the regulation of ATP hydrolysis leading to IFN response and self- and non-self-RNA discrimination could advance the development of effective therapeutic agents against autoimmune diseases.

## Introduction

Innate immune recognition of invading pathogens requires strict regulation for the discrimination of self- and non-self-antigens by pathogen recognition receptors (PRRs), such as retinoic acid inducible gene-I (RIG-I)-like receptors (RLRs). A family of RLR proteins, consisting of RIG-I, melanoma differentiation associated-5 (MDA5), and laboratory of genetics and physiology 2 (LGP2), recognizes double-stranded RNAs (dsRNAs) derived from replicating RNA viruses in the cytoplasm.

RIG-I preferentially selects dsRNA with 5′-triphosphate (5′ppp) at the end of the viral RNA (5′ppp dsRNA), and MDA5 selects a long dsRNA region at the viral RNA stem [1–4]. RIG-I and MDA5 form a filamentous oligomeric structure on viral dsRNA and trigger sophisticated signal transduction pathways through mitochondrial antiviral-signaling protein (MAVS) [1–4], which activates serine kinases that mediate activation of the transcription factors interferon regulatory factor-3 (IRF-3) and nuclear factor (NF)-kB [1–4]. These transcription factors are key regulators of type I interferon (IFN) and the expression of other immunostimulated genes required for the restriction of viral replication, cell death, and general immune responses [5].

Although RLR proteins exhibit similar structural architectures in their RNA-binding domains, such as the helicase and C-terminal domains, the structure of the third RLR protein, LGP2, is unique. LGP2 lacks the caspase activation and recruitment domain (CARD) required for signal transduction to MAVS and, therefore, cannot activate antiviral gene induction alone [6]. Another unique feature of LGP2 is its dual positive and negative regulatory roles in antiviral signaling and immune responses [7–10]. LGP2-deficient mice are more susceptible to picornavirus infections, such as that induced by encephalomyocarditis virus (EMCV), which is associated with MDA5 recognition [7,8]. This suggests that LGP2 facilitates the MDA5-mediated promotion of signal transduction to MAVS [7,8]. In contrast, the bone marrow-derived dendritic cells of LGP2-deficient mice [9] and LGP2-knockout human fibroblast cells exhibit marked increase in RIG-dependent immune responses [10]. These effects are indicative of a negative regulatory role of RIG-I in antiviral signaling.

Mechanistic analyses suggest that LGP2 stabilizes MDA5 RNA binding and the co-operative formation of ribonucleoprotein filaments for signal transduction and positive regulation [11]. In addition, LGP2 targets ubiquitin-conjugating enzyme 13 (UBC13)/ubiquitin-conjugating enzyme E2 N (UBE2N), thereby disrupting the K63-ubiquitin system [10]. These effects ultimately lead to the repression of NF-kB and IRF-3 signaling and negative regulation of the immune response [10].

While restricting viral replication is essential for immunity, accelerated or sustained IFN-stimulated antiviral gene expression is detrimental and contributes to autoimmune disorders [12], including systemic lupus erythematosus, Aicardi-Goutières syndrome, and Singleton-Merten syndrome [12]. Accordingly, immunoregulatory gene expression is tightly regulated by negative feedback mechanisms; additionally, non-self- and self-molecule discrimination is also tightly controlled. These regulatory mechanisms require the presence of redundant cofactors or systems that support the proper recognition of pathogen-associated molecular pattern molecules (PAMPs) or activation of immune signaling [1,13] and redundant negative regulators of immune responses [14–16].

The intrinsic ATP hydrolysis activity of RLRs is fundamental to discriminating between self- and non-self-RNA. As part of a proof-reading function, ATP hydrolysis of RIG-I dissociates self-RNA faster than it does 5′ppp dsRNAs and converts RIG-I to a signaling-active oligomeric state [17–20]. The ATP hydrolysis cycle mediated by MDA5 has also been suggested to have a proof-reading function that tests MDA5 interactions with bound RNA—ATP hydrolysis promotes the dissociation of MDA5 from loosely bound endogenous self-RΝΑ while maintaining bonds with viral dsRNAs, thus forming a filament structure for immune signal activation [21,22].

In addition to being involved in self- and non-self-RNA discrimination, RIG-I- and MDA5-induced ATP hydrolysis also contributes to displacing viral proteins prebound to dsRNA to assist antiviral proteins, including RNA-dependent protein kinase (PKR) and RIG-I for their RNA recognition [23]. Compared with that of RIG-I and MDA5, the biological role of LGP2-induced ATP hydrolysis is largely unknown, although it has been reported to be essential in the IFN response against picornavirus infection [8,24].

We previously reported that the RNA-silencing regulatory protein transactivation response RNA-binding protein (TRBP) enhances *Cardiovirus*-triggered immune responses through an association with LGP2, suggesting that TRBP is a cofactor for MDA5/LGP2-mediated immune response [24]. However, the underlying mechanism remains unknown. In the present study, we investigated the mechanism underlying TRBP-assisted regulation of immune responses and hypothesized that TRBP modulates LGP2-mediated ATP hydrolysis. To this end, we conducted a real-time ATP hydrolysis assay and monitored the effect of TRBP on RLR activity. Elucidation of the mechanism underlying the regulation of ATP hydrolysis leading to IFN response and self- and non-self-RNA discrimination could advance the development of effective therapeutic agents against autoimmune diseases.

## Materials and methods

### Plasmids, antibodies, and enzymes

The FLAG-tagged LGP2, RIG-I, MDA5, HA-tagged TRBP, and reporter plasmids used in the present study are described elsewhere [24]. Anti-FLAG M2 (1:2000; Cat#: F3165) and HA (1:2000: Cat#: H6908) antibodies were purchased from Sigma-Aldrich (St. Louis, MO, U.S.A.). TRBP (1:2000; Cat#: 15753-1-AP) and GAPDH (1:10000; Cat#: 60004-1-Ig) antibodies were purchased from proteintech (Rosemont, IL, U.S.A.). Lambda protein phosphatase was purchased from New England Biolabs (Cat#: P0753S, Ipswich, MA, U.S.A.). Phosphomimetic (SD) and nonphosphorylatable (SA) mutants of TRBP expression plasmids were constructed using the conventional QuickChange site-directed mutagenesis method (https://www.agilent.com/store/primerDesignProgram.jsp?toggle=uploadNow&mutate=true&_requestid=642015).

### Recombinant proteins and ATPase assay

Purified recombinant RLR proteins were prepared using 293FT cells, and the recombinant TRBP protein was prepared as previously described [24]. An ATPase assay was performed using the Enzcheck phosphate assay kit (Cat#: E6646, Thermo Fisher Scientific, Waltham, MA, U.S.A.) according to the manufacturer’s instructions, with slight modification. Briefly, 0.6–15 pmol recombinant RLR and TRBP proteins were mixed in 1× buffer, purine nucleoside phosphorylase (PNP), 2-amino-6-mercapto-7-methylpurine riboside (MESG), pIC (∼400 ng, InvivoGen), and 1 mM ATP in a 100 μl reaction volume in a 96-well plate on ice. The reaction was started at 37°C in a plate reader (Infinite M200PRO, Tecan), and the absorbance change at 360 nm was monitored every 60 s for 2 h. The ATP hydrolysis reaction speed was calculated as the linear point in the initial velocity of the reaction and indicated as the released phosphoric acid (Pi) concentration (mM) per second. The initial reaction velocity is estimated by determining the increased absorbance per fixed time at the linear reaction phase (2000–3000 s) in a 96-well culture plate, which can accommodate multiple samples.

### Cells and virus

The 293FT cells were purchased from Thermo Scientific (Cat#: R70007) and cultured in Dulbecco’s modified Eagle’s medium (DMEM, Cat#: D5796, Sigma-Aldrich) supplemented with 10% fetal calf serum (Hyclone, Marlborough, MA, U.S.A.) and penicillin/streptomycin (Cat#: 15140122, Thermo Scientific). EMCV was purchased from ATCC (VR-129B).

### Reporter gene assays

The 293FT cells were seeded onto a 48-well plate (4–6 × 10^4^; 70–80% confluency) and transfected with p3xFLAG-CMV10 empty vector or p3xFLAG-LGP2 (20 ng) with pEFBOS-FLAG-MDA5 (20 ng), pcDNA3HA-TRBP (20–200 ng), IFNβ-110 luc (125 ng), pRLnull (25 ng) (Promega, Madison, WI, U.S.A.), and other plasmids in the legend using Lipofectamine 3000. For viral infection, the transfected cells were incubated with EMCV (multiplicity of infection [MOI] = 1) in DMEM, supplemented with 2% serum, for 2 h; then, complete DMEM was added, followed by incubation for 14–17 h. We used a dual-luciferase reporter assay system (Promega) for luciferase assays and *Renilla* luciferase as the internal control to normalize luciferase activity.

### Gel-shift assay

A sense strand 45-mer RNA (5′-UUACUAUUGACACUAUGGCUUGCAAACAUG GAUACCCAGAUGUGU-3′) and an antisense strand 45-mer RNA (5′-ACACAUCUGG GUAUCCAUGUUUGCAAGCCAUAGUGUCAAUAGUAA-3′) from the Theiler’s murine encephalomyelitis virus genomic sequence were synthesized by Japan Bio Services (Saitama, Japan) and annealed for dsRNA preparation. The dsRNA (60 nM) was incubated with recombinant TRBP wild-type (WT) or TRBP SD protein (0, 12.5, 25, 50, 100, 200, or 400 pmol) in buffer A (20 mM HEPES [pH 7.5], 150 mM NaCl, 1.5 mM MgCl_2_, and 2 mM DTT) at 30°C for 30 min. The dsRNA-TRBP complex was separated on a 5% acrylamide gel and stained with SYBR Gold (Thermo Scientific). Relative RNA binding was quantified as the ratio of the shifted and original RNA band intensities using the Quantity One software (Bio-Rad Laboratories).

### Statistical analysis

All experiments, including the luciferase and ATP hydrolysis assays, were performed independently at least three times with similar results. Differences between groups were assessed using Student’s *t*-test. Significance was set at *P*<0.05.

## Results

### LGP2-induced ATP hydrolysis and RNA-binding are required for TRBP-mediated EMCV-triggered IFN promoter activation

We previously reported that the RNA-silencing regulatory protein TRBP positively regulates *Cardiovirus*-triggered type I IFN responses in small-interfering RNA (siRNA) knockdown and TRBP-overexpression experiments [24].

**Figure 1:**
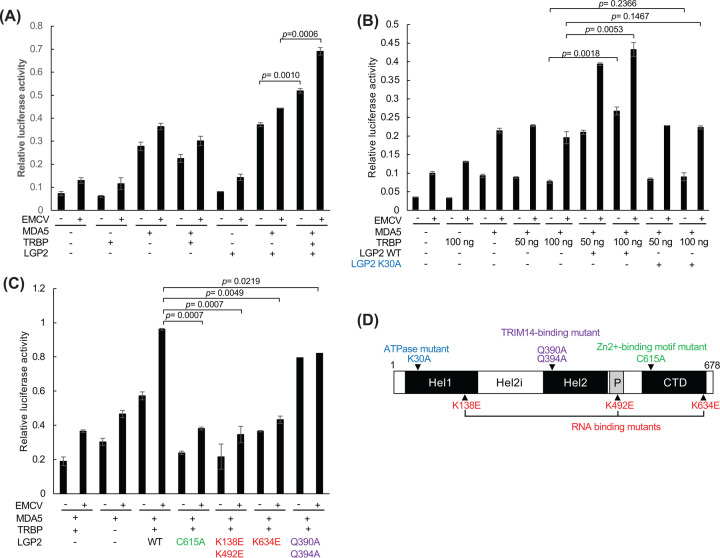
ATP hydrolysis and RNA-binding activity of LGP2 are required for TRBP-mediated IFNβ promoter up-regulation 293FT cells seeded onto a 48-well plate were transfected with MDA5 with WT LGP2 (**A**), ATPase-deficient LGP2 (K30A) (**B**), or other LGP2 mutants (**C**), and TRBP expression vectors with the −110 luc IFNβ promoter vector and pRL-SV40 and finally infected (+) or mock infected (−) with EMCV (MOI = 1) for 14–17 h. Cell lysates were analyzed for dual-luciferase activity (*n*=3). Differences between groups were assessed using Student’s *t*-test, with significance set at *P*<0.05. (**D**) Schematic representation of LGP2 structure and mutants.

The reporter gene assay confirmed the TRBP-mediated *Cardiovirus*-triggered IFNβ promoter activity in 293FT cells. MDA5 alone enhanced both basal and EMCV-induced IFN response, and addition of LGP2 (MDA5/LGP2) further enhanced this activity compared with that in the control (Lanes 5, 6, 11, and 12; Figure 1A). TRBP and LGP2 did not show significant effects (Lanes 2, 3, 8, and 9; Figure 1A). Coexpression of MDA5 and LGP2 in mock or EMCV-infected 293FT cells induced the TRBP-dependent enhancement of IFNβ promoter activity, which was not observed with MDA5 alone (Lanes 5–8, 11–14; Figure 1A). This finding is consistent with that in our previous report [24]. Several studies have reported that the ATP hydrolysis activity of LGP2 is required for *IFNβ* gene induction triggered by picornavirus infection [8,25,26]. Therefore, we tested whether LGP2-induced ATP hydrolysis is necessary for TRBP-mediated enhancement of IFNβ promoter activity. TRBP enhanced IFNβ promoter activity in mock and EMCV-infected cells expressing MDA5 and WT LGP2 but not in those expressing MDA5 and mutant LGP2 (K30A) with deficient ATP hydrolysis activity (Lanes 11–18, Figure 1B). These results suggest that the ATP hydrolysis activity of LGP2 is required for the TRBP-mediated enhancement of IFNβ promoter activity.

Other mutations in LGP2 residues critical for RNA binding (K138A/K492A and K634E) and a cysteine residue in the C-terminal domain crucial for binding to the dsRNA-binding protein PACT and Zn^2+^ ion (C615A) also abolished TRBP-induced IFN responses in mock or EMCV-infected cells (Figure 1C). Mutations of the a3 helix of the Hel2 domain (Q390A/Q394A), which abolish the interaction between LGP2 and TRIM14 [27], did not affect the virus-induced IFN response, whereas the basal IFN response in mock infected cells increased (Figure 1C).

All these results suggest that the ATP hydrolysis and RNA binding of LGP2 are required for TRBP to up-regulate MDA5-mediated type I IFN induction.

### Phosphomimetic TRBP modestly enhances IFNβ promoter activity

**Figure 2:**
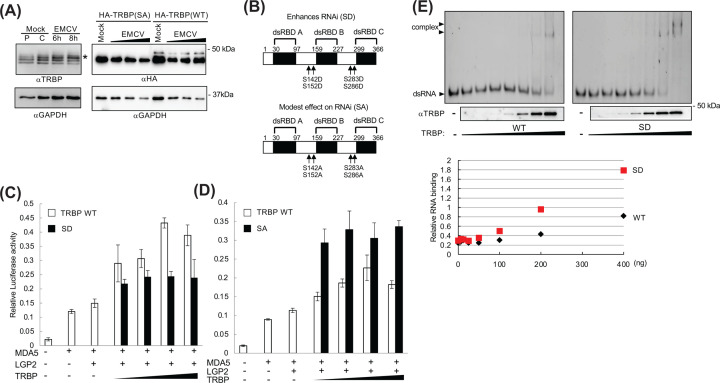
Phosphorylation status of TRBP impacts the LGP2/MDA5-dependent IFNβ promoter activity induced by EMCV infection (**A**) (Left) L929 cells were mock-infected or EMCV-infected (MOI = 1) for the indicated time period. Cell lysates were subjected to western blot analysis using anti-TRBP and GAPDH antibodies. The control mock infected cell lysate (C) was treated with lambda phosphatase to assess the phosphorylation status (P). (Right) 293FT cells transfected with HA-tagged TRBP (WT) or nonphosphorylatable TRBP (SD) vector were mock- or EMCV infected (MOI = 0.5–5). Cell lysates were subjected to western blot analysis using anti-HA and GAPDH antibodies. The asterisk indicates phosphorylated TRBP. (**B**) Schematic representation of TRBP structure and mutants. (**C,D**) 293FT cells were transfected with an empty vector, MDA5 and LGP2 expression vectors, and WT, phosphomimetic (SD), or nonphosphorylatable TRBP (SA) expression vector with the 110 luc IFNβ promoter vector and pRL-SV40 and finally infected with EMCV (MOI = 1) for 14–17 h. Cell lysates were analyzed using a luciferase assay (*n*=3). (**E**) The dsRNA-TRBP complex was separated on a 5% acrylamide gel, followed by staining with SYBR-Gold. A portion of the complex was analyzed via western blot analysis with anti-TRBP antibody. Relative RNA binding was quantified (below) as the ratio of the shifted and original RNA band intensities.

TRBP has four known phosphorylation sites at serine residues; the phosphorylation of TRBP enhances its activity for microRNA (miRNA) production and miRNA-targeted silencing [28]. In addition, activation of a kinase responsible for TRBP phosphorylation, mitogen-activated protein kinase (MAPK), has been reported to enhance the replication of EMCV in L929 cells [29]. Here, we first detected endogenous TRBP protein to confirm its phosphorylation upon EMCV infection using L929 cells. TRBP showed multiple bands representing multiple phosphorylation sites, which disappeared with phosphatase treatment of the control sample (Lanes P and C, Figure 2A). Upon EMCV infection, the ratio of higher molecular weight bands to lower molecular weight bands was increased in time-dependent manner, suggesting EMCV-triggered phosphorylation of TRBP via activation of protein kinase(s) (Figure 2A, left).

Next, we examined the potential effect of TRBP phosphorylation on *Cardiovirus*-triggered IFN induction. Expression plasmids for phosphomimetic and nonphosphorylatable TRBP mutants with all four serine residues replaced with aspartic acid (SD) and alanine (SA), respectively, were generated (Figure 2B). Higher molecular weight bands, which are believed to correspond to phosphorylated TRBP, were observed in cells expressing WT TRBP under mock- and EMCV-infected conditions, in contrast with cells expressing the SA mutant, where these bands were absent, alongside the main TRBP bands (Figure 2A, right).

The IFNβ promoter activity was analyzed using these mutant plasmids for EMCV-infected cells. While both mutants retained positive regulatory activity (Figure 2C,D, Lanes 3 and 4), TRBP SD showed reduced activity and TRBP SA exhibited hyperactivity in enhancing the EMCV-triggered IFNβ promoter activity compared with TRBP WT (Figure 2C,D).

We compared the RNA binding of TRBP SD and the nonphosphorylated TRBP WT using the gel-shift assay with a 45-mer synthetic dsRNA derived from Theiler mouse encephalomyelitis virus. Incubation of RNA with increasing amounts of recombinant TRBP indicated that TRBP SD had higher levels of RNA-binding activity than nonphosphorylated TRBP did (Figure 2E).

### Real-time monitoring of ATP hydrolysis reveals different characteristics of RLRs

**Figure 3:**
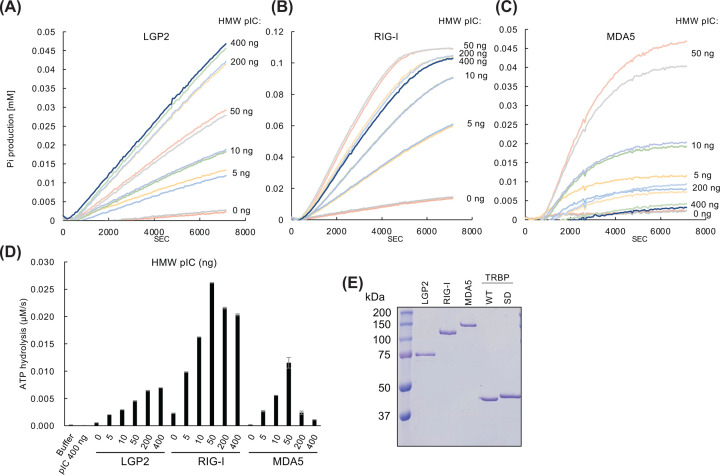
Real-time measurement of the RLR-induced RNA-dependent ATP hydrolysis (**A**) Recombinant LGP2, (**B**) RIG-I, or (**C**) MDA5 was incubated with the indicated amount of HMW pIC and 1 mM ATP in duplicate. The released Pi reacted with the substrate and was continuously monitored by measuring the absorbance at 360 nm for up to 7000 s using a spectrophotometer. (**D**) ATP hydrolysis velocity was calculated by determining the increased absorbance at the linear reaction phase (1500–4000 s); error bars indicate mean (*n*=2) ± standard deviation. (**E**) Recombinant proteins used in ATP hydrolysis and gel-shift assays; 200 ng LGP2, RIG-I, MDA5, nonphosphorylated TRBP (WT), and phosphomimetic TRBP (SD) were electrophoresed via SDS-PAGE and stained with Coomassie brilliant blue.

We analyzed RLR-mediated ATP hydrolysis using a colorimetric real-time ATP hydrolysis assay, which detected both the reaction velocity and persistence of ATP hydrolysis. This assay monitors Pi release in real-time for up to 7000 s, whereas most previously reported assays determined the activity according to the reaction endpoint [17–19]. All RLRs had a linear reaction phase in the production of Pi for up to 2000–3000 s, following polyinosinic-polycytidylic acid (pIC) stimulation. MDA5 showed a relatively early decline in Pi production when stimulated with high-molecular weight (HMW) pIC (Figure 3D), whereas LGP2 showed a constant increase for up to 7000 s.

Increasing the HMW pIC amount by up to 400 ng stimulated the LGP2-induced reaction velocity in a dose-dependent manner (Figure 3A). The RIG-I-induced reaction velocity was saturated by the addition of 50 ng pIC (Figure 3B,D) but increased in a dose-dependent manner with the addition of up to 50 ng HMW pIC. The MDA-5-induced reaction velocity sharply declined with increasing amounts of HMW pIC (Figure 3C,D). Low-molecular weight (LMW) pIC stimulation showed a similar pattern, but a sharp decline in MDA5 activity with higher amounts of pIC was not observed (Supplementary Figure 1).

### TRBP enhances RNA-dependent LGP2-induced ATP hydrolysis and differentially regulates RLRs in the presence and absence of RNA

**Figure 4:**
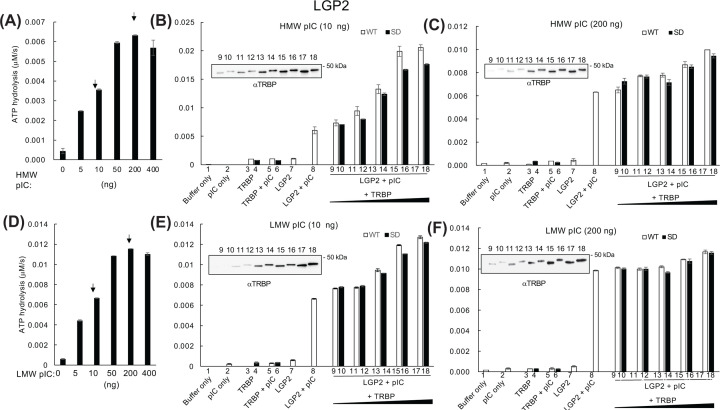
Regulation of LGP2-induced ATP hydrolysis by TRBP Effect of increasing concentrations (0–400 ng) of (**A**) HMW pIC or (**D**) LMW pIC on the LGP2-induced ATP hydrolysis. Arrows indicate the amounts of pIC used in (**B,C,E,F**). LGP2 (0.66 pmol) was incubated with phosphomimetic (SD) or WT TRBP (0.6–15 pmol) with 10 or 200 ng (**B,E**) HMW or (**C,F**) LMW pIC, and ATP hydrolysis was measured. Buffer, pIC, LGP2, or TRBP-only controls (Lanes 1–4 and 7) and LGP2 or TRBP with pIC controls (Lanes 5, 6, and 8) were also included. To ensure equal loading of TRBP, assay samples were analyzed via western blotting with TRBP antibody. Error bars indicate mean (*n*=2) ± standard deviation.

Given the important role of LGP2-induced ATP hydrolysis in TRBP-mediated IFNβ promoter activation, we assessed whether TRBP modulates this effect using the real-time ATP hydrolysis assay. pIC-dependent LGP2-induced ATP hydrolysis was enhanced by TRBP (1:1 to 1:23 molar ratio against LGP2) in a dose-dependent manner (Figure 4) with both lower (10 ng) and higher (200 ng) amounts of HMW (Figure 4A–C) and LMW (Figure 4D–F) pIC.

**Figure 5:**
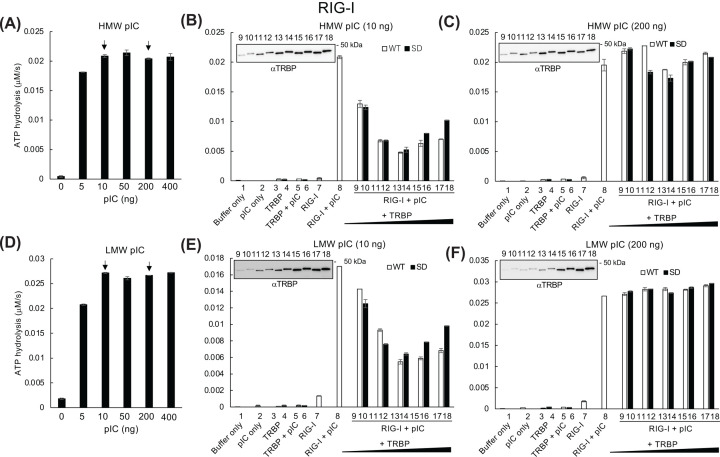
Regulation of RIG-I -induced ATP hydrolysis by TRBP Effect of increasing concentrations (0–400 ng) of (**A**) HMW pIC or (**D**) LMW pIC on the RIG-I-induced ATP hydrolysis. RIG-I (0.66 pmol) was incubated with phosphomimetic (SD) or WT TRBP (0.6–15 pmol) with (**B,E**) 10 ng or (**C,F**) 200 ng HMW or LMW pIC, and ATP hydrolysis was measured as in Figure 4.

**Figure 6:**
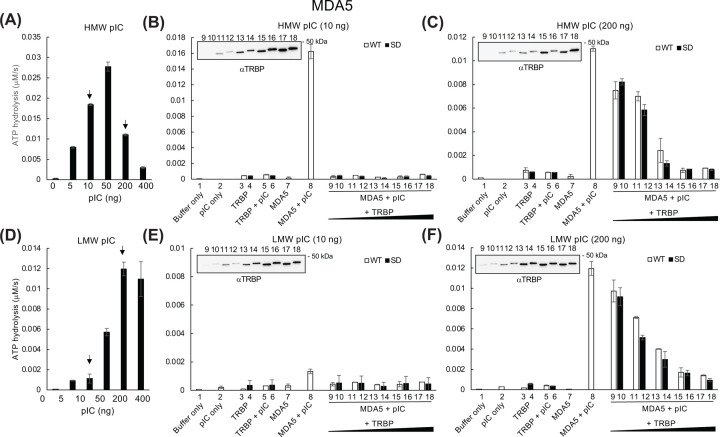
Regulation of Regulation of MDA5-induced ATP hydrolysis by TRBP Effect of increasing concentrations (0–400 ng) of (**A**) HMW pIC or (**D**) LMW pIC on the MDA5-induced ATP hydrolysis. MDA5 (1 pmol) was incubated with phosphomimetic (SD) or WT TRBP (0.6–15 pmol) with 10 or 200 ng (**B,E**) HMW or (**C,F**) LMW pIC, and ATP hydrolysis was measured as in Figure 4.

Because the reporter gene assay (Figure 2) showed that the difference in IFNβ promoter activation depended on TRBP phosphorylation status, we examined the effect of phosphomimetic TRBP on ATP hydrolysis. Consistent with the results of IFNβ promoter reporter gene assay, nonphosphorylated WT TRBP induced slightly higher LGP2-mediated hydrolysis activity than phosphomimetic TRBP did with both lower and higher levels of HMW or LMW pIC stimulation. In contrast, RIG-I-mediated ATP hydrolysis with lower amounts of TRBP (0.6–5 pmol) was inhibited, whereas higher amounts (10–15 pmol) reversed this trend after stimulation with a lower amount (10 ng) of pIC. TRBP did not affect RIG-I activity stimulated with a higher amount (200 ng) of HMW or LMW pIC (Figure 5). MDA5-induced ATP hydrolysis was strongly inhibited by TRBP under all conditions (Figure 6).

**Figure 7:**
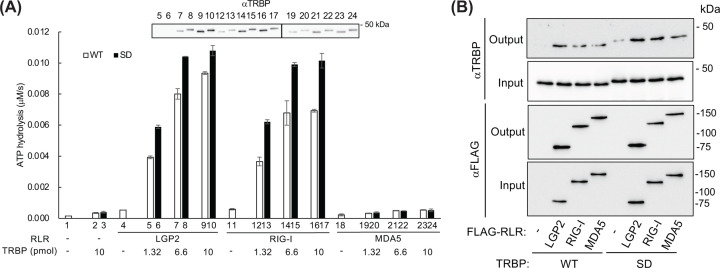
Regulation of RLR-induced ATP hydrolysis by TRBP without RNA (**A**) LGP2 (0.66 pmol), MDA5 (1 pmol), or RIG-I (0.66 pmol) was incubated with phosphomimetic (SD) or WT TRBP (0.6–15 pmol) without pIC, and ATP hydrolysis was measured as in Figure 4. (**B**) LGP2 (150 ng), MDA5 (150 ng), or RIG-I (150 ng) was subjected to incubation in the presence of either phosphomimetic (SD) or WT TRBP (50 ng), utilizing a whole-cell extract buffer (50 mM Tris-HCl [pH 8.0]-280 mM NaCl-0.5% NP-40-0.2 mM EGTA-2 mM EDTA-1 mM dithio-threitol-1 mM MgCl_2_). TRBP in isolation, in the absence of any RLR protein, was utilized as a negative control (−). The FLAG-RLR proteins were isolated using FLAG agarose beads, and the captured RLRs were subsequently assessed alongside co-purified TRBP through western blot analysis, utilizing both FLAG and TRBP antibodies to evaluate both the input mixture prior to purification and the resulting output.

We also tested the effect of TRBP on RLR-mediated ATP hydrolysis in the absence of RNA (Figure 7A). Surprisingly, TRBP enhanced the LGP2-induced ATP hydrolysis. In contrast with pIC-stimulated conditions, phosphomimetic TRBP more dominantly enhanced LGP2-induced ATP hydrolysis than the nonphosphorylated WT did. Similarly, TRBP also enhanced the activity of RIG-I in the absence of RNA, with phosphomimetic TRBP exhibiting the highest level of enhancement; however, TRBP did not show any effect on MDA5. The evidence suggests that TRBP is capable of directly regulating the ATP hydrolysis process of RLRs, independent of RNA presence or phosphorylation state of TRBP. To validate this, immunoprecipitation experiments were conducted using recombinant proteins from the ATP hydrolysis assay, confirming a direct interaction between TRBP and RLRs. Notably, nonphosphorylated TRBP (WT) and SD-TRBP were co-purified with RIG-I and LGP2. Moreover, an interaction between TRBP and MDA5 was also observed (Figure 7B).

## Discussion

In the present study, we investigated the mechanism underlying the effect of the RNA-silencing regulatory protein TRBP on MDA5/LGP2-induced IFN signaling. Our results demonstrated that TRBP requires LGP2-mediated ATP hydrolysis and RNA-binding for up-regulating the MDA5/LGP2-mediated IFNβ promoter activity.

Since the phosphorylation status of TRBP impacts miRNA regulation [28] and EMCV activates the kinase responsible for TRBP phosphorylation for virus replication [29], we examined the effect of phosphomimetic TRBP on IFN signaling. Our findings suggested that phosphomimetic TRBP had a modest effect, whereas nonphosphorylatable TRBP exhibited higher activity than TRBP WT in enhancing the IFN response against EMCV infection, indicating that EMCV attenuates the TRBP-mediated IFN response via phosphorylation of TRBP as an immune evasive strategy for replication. In addition, this effect was in contrast with that observed in the miRNA processing and silencing mediated by TRBP [28].

TRBP is a dsRNA-binding protein that interacts with Dicer, a multidomain enzyme that consists of several RNA-binding domains, to enhance the processing of pre-miRNA into miRNA duplexes. TRBP is suggested to enhance the processing of a subset of miRNAs that bind to it through a miRNA-binding preference [30–32]. In addition to our study on the TRBP-LGP2 association, the close relationship between RNA silencing and the IFN system has been described previously [32–34]. Takahashi et al. [32] demonstrated that LGP2 inhibits pre-miRNA binding and recruitment by TRBP, thereby repressing specific miRNA activities. Van der Veen et al. [33] reported that LGP2 associates with Dicer to inhibit Dicer-dependent processing of long dsRNA and block RNAi in mammalian cells.

The major antiviral system of mammalian innate immunity is the protein-based IFN system. In contrast, the primary antiviral defense strategy of plants and invertebrates is RNA-based antiviral immunity, wherein virus-derived siRNAs are loaded onto the RNA-induced silencing complex (RISC) and target complementary viral RNA to block virus replication. However, recent studies have suggested that small RNA-mediated antiviral systems are also active in mammals, depending on the cell type [35–37], although the IFN- and RNA-silencing systems suppress each other [38,39]. The phosphorylation status of TRBP might have some implications in the reciprocal inhibition between IFN signaling and the RNAi machinery in mammalian cells. Alternatively, the phosphorylation status could control the balance between the IFN- and RNA-silencing systems, although further investigation is necessary.

LGP2-induced ATP hydrolysis is a requirement for the picornavirus-mediated IFN response [8,25,26] and the present study revealed that ATP hydrolysis is required for TRBP-dependent IFN up-regulation. Consequently, we hypothesized that TRBP up-regulates LGP2-induced ATP hydrolysis to enhance the IFN response. To assess this hypothesis, we developed a real-time ATP hydrolysis assay for RLRs and initially examined RLR-mediated ATP hydrolysis. We observed the differences in duration of hydrolysis activity among RLRs and found that LGP2 and RIG-I showed longer durations, whereas MDA5 showed an early decline in activity, especially with HMW pIC. This indicates that continuous pIC stimulation structurally transforms MDA5 to the inhibitory state for ATP hydrolysis. Because MDA5- or RIG-I-induced ATP hydrolysis contributes to the discrimination of self- and non-self-RNAs, sustained MDA5-induced ATP hydrolysis might be more detrimental to self-RNA surveillance than that induced by RIG-I. In fact, self-RNAs are susceptible to being considered as viral RNAs by MDA5 rather than by RIG-I, unless self-RNAs are properly processed by adenosine deaminase acting on RNA 1 (ADAR1) [40–42]. We noted that TRBP not only up-regulated the MDA5/LGP2-mediated IFN response to infection but also the basal IFN response in uninfected cells (Figure 1). This suggests that TRBP enhances the self-RNA recognition mediated by overexpressed MDA5/LGP2. In 293FT cells, transfection of MDA5 itself efficiently enhanced IFN promoter activity (Figure 1). It was reported that gain-of-function MDA5 mutants expressed in HEK293T cells recognize self-RNA, thereby causing oligomerization of CARDs, which triggers downstream signaling for the IFN response [43]. If WT MDA5 up-regulates IFN in a similar manner when overexpressed, TRBP may contribute to sensitization of self-RNA recognition by MDA5 through interaction with LGP2.

We evaluated whether the RNA binding of TRBP is required for the up-regulation of ATP hydrolysis activity using mutants with possible defects in RNA binding (K80A, K210A, and K80/210A) [44], since RNA-independent enhancement of ATP hydrolysis of RIG-I and LGP2 by TRBP was observed (Figure 7). While K80A mutant retained the RNA-binding activity, K210A or K80/210A showed reduced RNA binding in the gel-shift assay (Supplementary Figure 2). The ATP hydrolysis activity of LGP2 was not up-regulated by the RNA-binding defect mutants, K210A or K80/210A, while it was activated by K80A mutant similarly as by WT TRBP (Supplementary Figure 3). The prominent effect by the RNA-binding defect mutants was not observed in the ATP hydrolysis of RIG-I and MDA5 (Supplementary Figure 3). These results suggested that the RNA-binding activity of TRBP is required for up-regulation of ATP hydrolysis activity of LGP2.

Using our real-time ATP hydrolysis assay, we demonstrated that TRBP positively enhanced the RNA-dependent ATP hydrolysis activity of LGP2 and, in parallel to IFN enhancement, phosphomimetic TRBP showed a lower effect than non-phosphorylated TRBP did. In contrast with its effect on LGP2, TRBP partially and strongly inhibited the RIG-I- and MDA5-induced RNA-dependent ATP hydrolysis, respectively, indicating that TRBP differentially regulates the activity of RLRs. Because TRBP is a negative regulator of RIG-I signaling [44], down-regulation of RNA-dependent ATP hydrolysis could be involved in the inhibitory mechanism. It is surprising that TRBP strongly inhibits the RNA-dependent ATP hydrolysis of MDA5 despite it being essential for MDA5 activation. This may mirror the slight inhibition of IFN promoter activity by TRBP when MDA5 alone is expressed; contrastingly, this activity is enhanced when MDA5 and LGP2 are coexpressed (Lanes 5–8, Figure 1A; Lanes 5–10, Figure 1B). Alternatively, TRBP might maintain the oligomeric state of MDA5 on dsRNA by inhibiting ATP hydrolysis [45].

It is intriguing that TRBP differentially regulates RLRs depending on the presence or absence of RNA. TRBP up-regulated the activity of RIG-I and LGP2 in the absence of RNA, while it partially inhibited RIG-I and activated LGP2 in the presence of RNA. Drawing on this observation, we have substantiated the interaction between RLRs and TRBP through the utilization of purified proteins (Figure 7B). The entirety of the outcomes suggest that TRBP governs the ATP hydrolysis process of RLRs through direct interaction. This finding contradicts our prior report, which indicated that TRBP interacted with LGP2 but not with RIG-I/MDA5 in immunoprecipitation experiments. [24]. This may be attributed to differences in experimental conditions, such as disparities in buffer composition and variations in the utilization of expressed proteins present in cell lysates versus their purified recombinant proteins.

TRBP may contribute to the structural transformation of RIG-I and LGP2 to their active forms for ATP hydrolysis independent of RNA presence. The up-regulation of ATP hydrolysis activity in RIG-I, which occurs independently of RNA, has the potential to result in the constitutive activation of IFN by TRBP under certain conditions.

Another dsRNA-binding protein, protein activator of interferon-induced protein kinase PACT (EIF2AK2 and PRKRA), which is structurally similar to TRBP, has also been reported to activate RIG-I-induced ATP hydrolysis in the absence of RNA, thereby enhancing the RIG-I-dependent antiviral response [46].

It is also interesting that the effect of phosphomimetic TRBP on the ATP hydrolysis activity of LGP2 varied with the absence or presence of RNA. Our RNA-binding experiments demonstrated that the TRBP SD mutant had a greater RNA-binding activity than the nonphosphorylated TRBP did (Figure 2D). Therefore, we speculate that RNA binding affects the ATP hydrolysis activity—a stronger RNA binding by TRBP SD would outcompete LGP2, resulting in weaker ATP hydrolysis—and that the SD mutant has a stronger effect in transforming LGP2 and RIG-I to the ATP hydrolysis forms in the absence of RNA than that in its presence. Nonetheless, it appears that TRBP mutants lacking RNA-binding ability have a negative impact on the ATP hydrolysis of LGP2 (Supplementary Figure 3), indicating that the RNA-binding property of TRBP is essential for this positive effect. This observation implies that the up-regulation of ATP hydrolysis requires the appropriate RNA-binding affinity of TRBP in the TRBP/LGP2/RNA complex.

The strong inhibition of MDA5 and weak inhibition of RIG-I during RNA-dependent ATP hydrolysis activity might be due to the sequestration of RNA by TRBP according to the order of RNA-binding activity (LGP2 > RIG-I > MDA5) [47].

**Figure 8:**
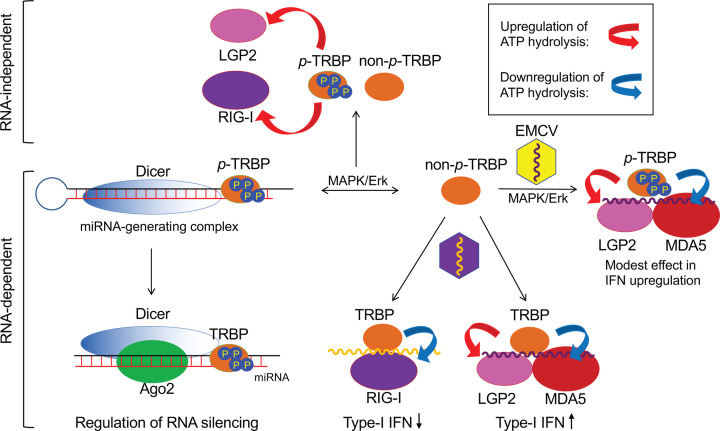
Model of TRBP-mediated differential regulation of the ATP hydrolysis activity of RLRs, IFN response, and RNA silencing TRBP phosphorylated by MAPK/ERK (p-TRBP) has enhanced activity in RNA silencing [28], while nonphosphorylated TRBP (non-p-TRBP) has enhanced activity in MDA5/LGP2-mediated IFN response. Since EMCV activates a kinase responsible for TRBP phosphorylation, MAPK, for virus replication [29], EMCV may attenuate the TRBP-mediated IFN response via TRBP phosphorylation. TRBP up-regulates the ATP hydrolysis activity of LGP2 and down-regulates that of MDA5 and RIG-I in the presence of RNA (HMW pIC), wherein TRBP positively and negatively regulates the MDA5/LGP2- and RIG-I-mediated IFN response, respectively. In addition, TRBP up-regulates the ATP hydrolysis activity of LGP2 and RIG-I, but not that of MDA5, in the absence of RNA.

The biological role of ATP hydrolysis modulation by dsRNA-binding proteins, such as TRBP and PACT, remains unclear. More specifically, we could not reveal the biological context of LGP2-mediated ATP hydrolysis and how the modulation of ATP hydrolysis contributes to the IFN response. However, we showed that TRBP up-regulated the ATP hydrolysis activity of LGP2, which is required to regulate the IFN response, and, in the phosphorylated state, differentially regulated RLRs depending on the presence of RNAs (Figure 8). Further research of the mechanism underlying the regulation of ATP hydrolysis that leads to IFN response and self- and non-self-discrimination would contribute to developing therapeutic agents, such as antivirals and treatments, for autoimmune diseases.

## Supplementary Material

Supplementary Figures S1-S3

## Data Availability

All supplementary material and raw data for western blot analysis can be accessed here: (https://www.dropbox.com/sh/vsiisiq8mp92vhm/AADSh6BogPZH3RlctbnmNaHoa?dl=0).

## Abbreviations

5′ppp: 5′-triphosphate
CARD: caspase activation and recruitment domain
EMCV: encephalomyocarditis virus
HMW: high-molecular weight
IFN: interferon
IRF-3: interferon regulatory factor-3
LGP2: laboratory of genetics and physiology 2
LMW: low-molecular weight
MAPK: mitogen-activated protein kinase
MAVS: mitochondrial antiviral-signaling protein
MDA5: melanoma differentiation associated-5
MOI: multiplicity of infection
NF: nuclear factor
Pi: phosphoric acid
pIC: polyinosinic-polycytidylic acid
RIG-I: retinoic acid inducible gene-I
RLR: RIG-I like receptor
TRBP: transactivation response RNA-binding protein
WT: wild-type
