# Supplementary material for: Differential regulation of ATP hydrolysis of RIG-I-like receptors by transactivation response RNA-binding protein
Source: Biosci Rep. 2023 May 5;43(5):BSR20222152. doi: 10.1042/BSR20222152 (PMC10170298; doi:10.1042/BSR20222152)
Supplement: Supplementary Figures S1-S3 [file BSR-2022-2152_supp.pdf]

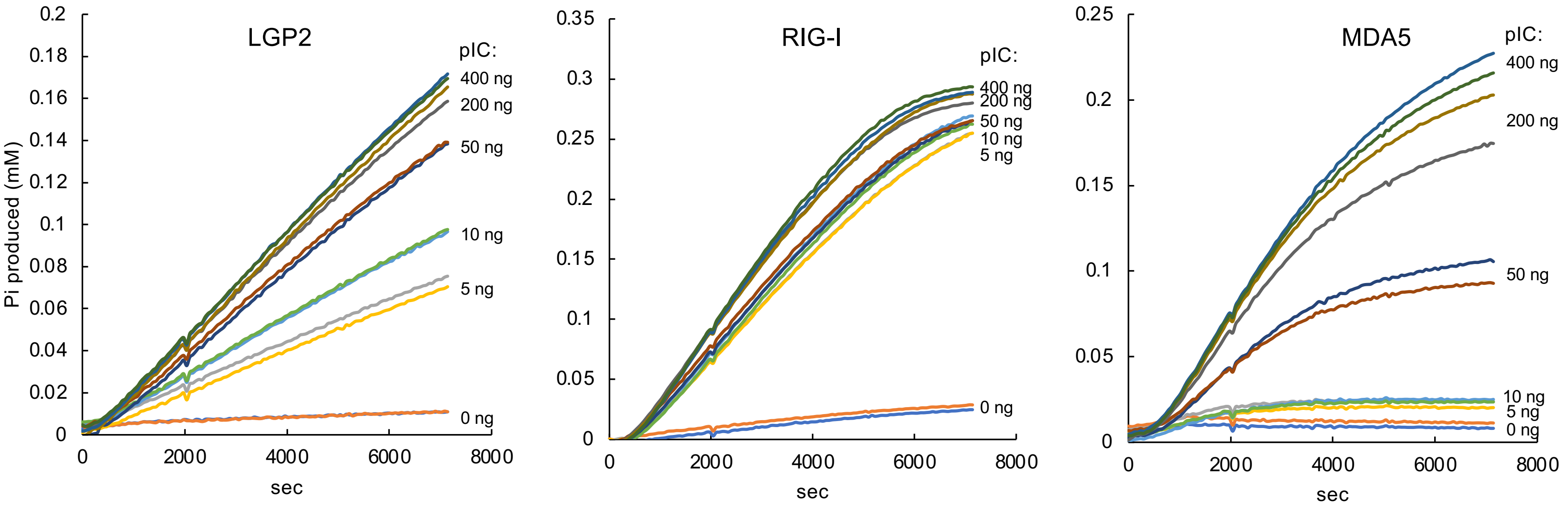

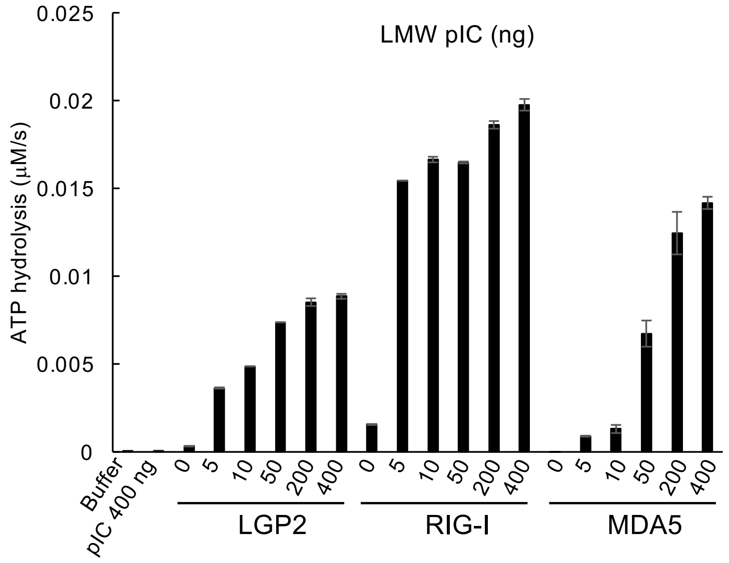

Supplementary Fig. 1 Realtime measurement of LMW pIC-dependent ATP hydrolysis activity of RLRs. Recombinant LGP2(A), RIG-I(B), or MDA5(C) was incubated with indicated amount of LMW pIC and 1 mM ATP. Released phosphoric acid (Pi) reacted with the substrate was continuously monitored by taking 360 nm absorbance as in Fig. 3. The velocity of ATP hydrolysis was calculated as in Fig. 3 (D).

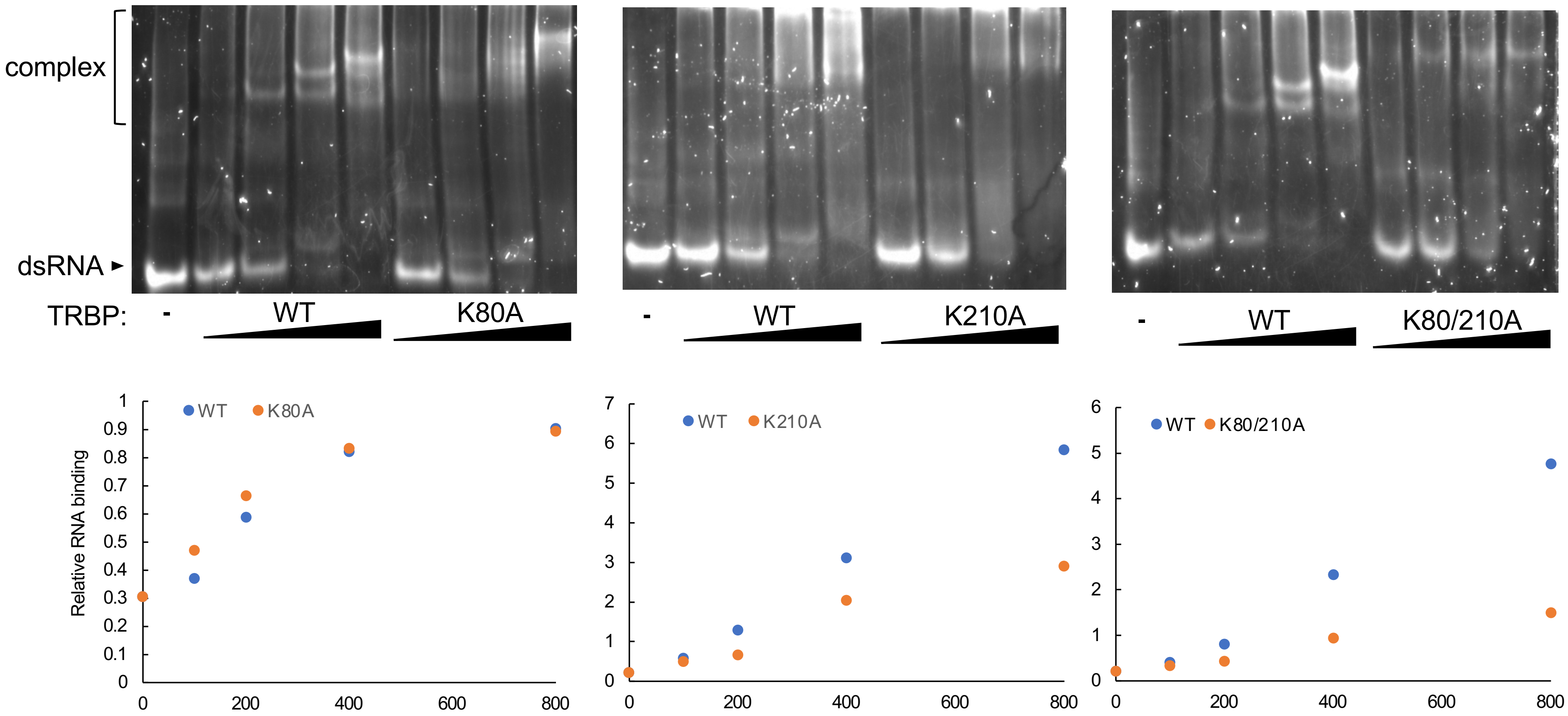

Supplementary Fig. 2 RNA binding activity of TRBP mutants. The dsRNA-TRBP complex was separated on acrylamide gels and relative RNA binding was quantified (below) as the ratio of the shifted and original RNA band intensities as in Fig. 2E.

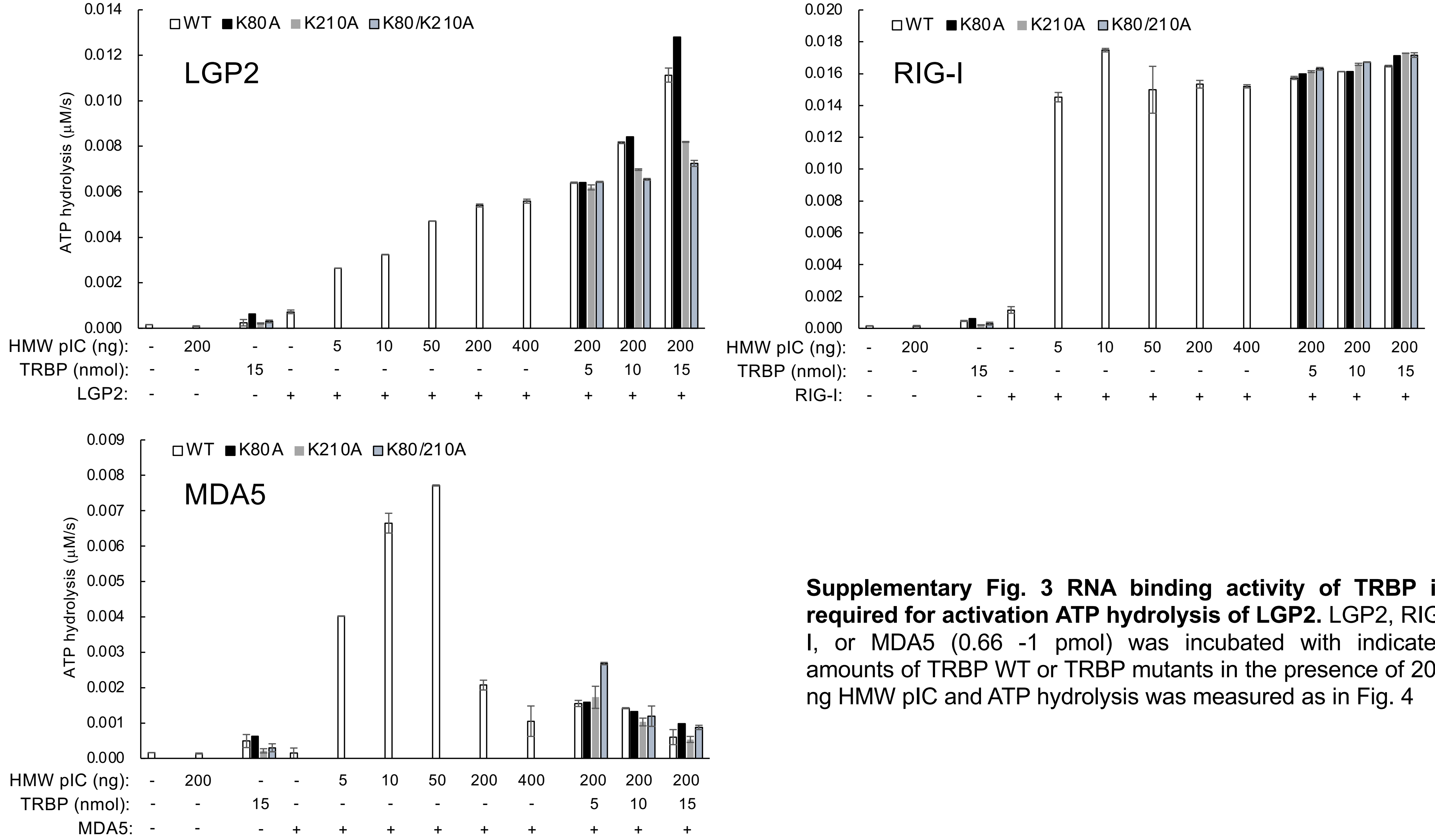

Supplementary Fig. 3 RNA binding activity of TRBP is required for activation ATP hydrolysis of LGP2. LGP2, RIG-I, or MDA5 (0.66 -1 pmol) was incubated with indicated amounts of TRBP WT or TRBP mutants in the presence of 200 ng HMW pIC and ATP hydrolysis was measured as in Fig. 4
